# A biochemical analysis of Black Soldier fly (*Hermetia illucens*) larval frass plant growth promoting activity

**DOI:** 10.1371/journal.pone.0288913

**Published:** 2023-07-19

**Authors:** Terrence Green

**Affiliations:** DipTerra LLC, Lake Oswego, OR, United States of America; Universita degli Studi della Basilicata, ITALY

## Abstract

Black Soldier fly (*Hermetia illucens*) larval (BSFL) frass was examined for its nutrient nitrogen, phosphate and potassium (N:P_2_0_5_:K_2_O), phytohormone and biogenic amine content, its plant growth promoting activity, and screened to test the hypothesis that bacteria characteristic of the genus *Enterococcus* (present in the biome of decaying catering waste and the larval gut) are excreted by BSFL in their frass. Frass plant growth promoting activity was measured by comparing the growth of winter wheat berry (*Triticum aestivum*) in frass treated soil to that of untreated (control) soil. The N:P_2_0_5_:K_2_O percent dry matter average, biogenic amine and phytohormone content of frass was determined by standard soil analysis, HPLC and HPLC/GC-MS methodologies, respectively. All were at too low of concentrations to account for its plant growth promoting activity. Frass added to soil induced a 11% increase in aerial mass and shoot length in treated plants over controls. Numerous colonies of *Enterococci* growing out on BEA (bile-esculin-agar) plates were detected in frass collected directly from larvae confirming the hypothesis that viable *Enteroccoci* are passing from the larval gut into their frass. Since a number of rhizobacteria, including *Enterococci*, have previously been identified as part of the larval gut biome, the passage of *Enterococci* from the larval gut into frass in the face of only trace N:P_2_0_5_:K_2_O percent dry matter averages, biogenic amine and phytohormone content is consistent with the hypothesis that *Enterococci* exhibiting rhizobacterial activity have a role in conferring to frass its plant growth promoting activity.

## Introduction

A number of researchers have conflated plant growth promoting activity associated with leftover waste residues recovered from Black Soldier fly (*Hermetia illucens*) larva (BSFL) with BSFL frass even though much of the waste residue, aside from frass, consists of waste feedstock in varying stages of degradation, insect exuviate, and microorganisms [1–8]. Frass, itself, has not heretofore been examined for its role in conferring to soil plant growth promoting activity, its N:P_2_0_5_:K_2_O percent dry matter average, its phytohormone and biogenic hormone content, or for rhizobacteria passed from the larval gut into frass which could also account for its plant growth promoting activity on amendment into soil.

Whereas it may be convenient from a commercial perspective to refer to waste residues left behind by BSFL as frass, the composition of larval processed waste is complex. Its composition varies depending on the source of the feedstock presented to larvae, how the feedstock is mixed and aerated, the age and degree of decomposition of the waste on amendment into soil, etc.

Plant growth promoting rhizobacteria, especially those associated with the genus *Enterococcus*, previously identified in decaying waste feedstocks and the larval gut of BSFL [9–11], and having the capability of colonizing the roots of receptive plants [12–16], are of particular interest concerning their possible role in conferring to frass its plant growth promoting properties. Fuhrmann *et al*. [17], furthermore, recently reported that microbial members of the genera *Bacillus* and *Streptomyces* of which rhizobacteria are also represented are present in the biome of BSFL processed spent brewer grain residues. These latter two genera have also been identified in the gut biome of BSFL [10, 11].

While there is presently no proof that rhizobacteria colonizing the guts of BSFL play a role in conferring to frass its growth promoting activity, the observations cited above suggest that there could be a link accounting for the beneficial effects of amending larval processed waste residues and frass into soil and the expression of plant growth promoting activity traced to beneficial rhizobacteria shed by larvae in their frass.

This study describes the plant growth promoting effect of BSFL frass recovered from BSFL processed catering waste residues on the growth of winter wheat berry seedlings grown in soil amended with frass, examines its biogenic amine, phytohormone, and N:P_2_0_5_:K_2_O percent dry matter average, and tests the hypothesis that *Enterococci* inhabiting the guts of BSFL are excreted in viable form in their frass.

BSFL frass was found to contain only trace amounts of N:P_2_0_5_:K_2_O, biogenic amines and phytohormones—all at far too low of concentrations to account for its plant growth promoting activity. Colonies of viable *Enterococci* were however found in frass collected directly from larvae. These latter findings confirm the hypothesis that *Enterococci* are excreted by larvae in viable form in their frass and lend further credence to the hypothesis that rhizobacteria may have a role in conferring to frass its plant growth promoting activity upon amending it into soil.

## Materials and methods

### Growth of BSFL and recovery of BSFL frass

BSFL were obtained by growing BSFL in bioreactors loaded every fourth day with catering waste obtained from discarded cafeteria kitchen waste. Frass was collected directly from larvae after they were washed and suspended in sterile water, and by slurring BSFL processed catering waste residues in tap water and filtering off particulates in the slurry through a 20 mesh stainless steel screen. Additional details on how the larvae and frass were obtained can be found in S1 File.

### Evaluation of BSFL frass plant growth promoting activity

Frass plant growth promoting activity was evaluated by measuring the effect of adding BSFL frass recovered from BSFL processed catering waste residues to potting soil in pots planted with winter wheat berry seedlings housed inside a greenhouse with diurnal exposure to natural sunlight and maintained at a constant temperature of 22°C. Seedlings grown in control (untreated) potting soil were grown in parallel with those grown in frass-treated potting soil. Control and frass-treated pots were placed adjacent to one another to ensure that all seedlings received the same light exposure throughout the course of the experiments. Frass-treated and control seedlings all received the same volume of water, and at the same frequency, throughout the course the experiments. In the experimental frass-treated test set, frass (50 ml—equivalent to the amount of frass recovered from 2.5 g dry weight BSFL processed residues) was delivered to each of four pots, each containing 2.5 g seeds per pot, one week after planting the seedlings in potting soil, and once again the following week. Four pots containing 2.5 g seeds per pot were set up at the same time and in the same manner to serve as controls. In this control group of plants, water instead of frass was added to the potting soil. After the second week of growth, pots containing both the control and frass treated seedlings received only water thereafter as needed through the remainder of the growth trial.

At the end of a 30-day growth interval, the upper plant masses of the frass-treated and control seedling sets of plants were harvested at soil level. The wet weight upper plant masses of the four frass-treated and corresponding control pots were each measured to the nearest 0.1 g from which the average wet weight mass ±1 standard deviation of frass-treated and control (untreated) seedlings was calculated.

The average shoot length ±1 standard deviation of the harvested plants grown in frass-treated and untreated (control) potting soil was also calculated by measuring to the nearest 0.1 cm the stem lengths of 137 shoots drawn at random after pooling and mixing the cuttings taken from all four pots for the frass-treated plants, and for the untreated control group of plants, respectively.

The statistical difference and significance between the frass-treated and control (untreated) plant outcomes in terms of upper aerial mass and stem length were calculated using the one-tailed student’s t-test.

### Detection of *Enterococci* discharged into frass

The detection of viable *Enterococci* excreted by BSFL feeding on catering waste was determined using BEA (bile-esculin-azide) agar plates streaked with wire loop samples drawn from aqueous suspensions of larvae washed free of catering waste as described in S1 File. BEA agar plates were incubated at 35°C and examined for colony growth 24 and 48 hours after plating out samples. Colonies growing on the surface of the agar plates accompanying generation of dark brown-black iron esculetin end product were interpreted as a positive test for viable *Enterococci* [18]. BEA agar plates were obtained from Hardy Diagnostics.

### Analysis of frass biogenic amine content

Leachate from catering waste unprocessed by larvae that drained through a 20 mesh stainless steel screen was analyzed for its biogenic amine content to establish a baseline level of biogenic amines for comparative purposes to the content of the same compounds measured in frass recovered from BSFL processed catering waste residues. The biogenic amines were all analyzed by Trilogy Analytical Laboratory, Washington, MO by HPLC [19].

### Analysis of frass phytohormone content

Leachate recovered from catering waste unprocessed by larvae that drained through a 20 mesh stainless steel mesh screen was analyzed for its phytohormone content to establish a baseline level of these plant regulatory compounds for comparative purposes to concentrations of the same compounds measured in frass. Phytohormones were analyzed by Lifesible, Shirley, NY, by HPLC-MS analysis. Details on the analysis method and results can be found in S2 and S3 Files.

### Analysis of frass and BSFL processed catering waste N, K_2_O and available P_2_O_5_ content

Replicate samples of frass separated from BSFL processed catering waste residues, and BSFL processed catering waste residues left behind in the BSFL bioreactors, were each analyzed for N, K_2_O and available P_2_O_5_ by A&L Western Agricultural Laboratory, Modesto, CA by AOAC Official Method 993.13 (combustion method), Method 983.02 (updated to flame emission spectroscopy in place of flame photometry) and Method 960.03 (phosphorous available in fertilizers), respectively, from which the N:P_2_0_5_:K_2_O percent dry matter average of each ±1 standard deviation was calculated.

### Statistical analysis of data sets

Statistical analysis of data sets in calculating the average of replicate samples, standard deviation (SD) and one-tailed t-tests were done using Microsoft Office 365 Excel, Version 2207 software.

## Results

Only trace amounts of cadaverine, histamine, putrescine and tyramine were detected in catering waste leachate. Phenylethylamine, spermidine, spermine and tryptamine were not detected in the leachate (**Table 1**). No biogenic amines were detected in frass.

**Table 1:** Comparison of biogenic amine and phytohormone content of catering waste leachate versus BSFL frass recovered from larval-processed catering waste residues.

***Biogenic amines (ng/ml)***	**Catering Waste Leachate**	**BSFL Frass**				
Cadaverine	7.1	<5 ^a^				
Histamine	13.7	<5 ^a^				
Putrescine	86.8	<5 ^a^				
Tyramine	9.5	<5 ^a^				
phenylethylamine, spermadine, spermine, tryptamine	<5 ^a^	<5 ^a^				
***Phytohormones (ng/ml)***						
indoleacetic acid	50.4^b^	0.36				
absicic acid	20.3 ^b^	0.04				
jasmonic acid	4.7 ^b^	0.18				
methyl jasmonate	0.14 ^b^	0.02				
giberrellin A1	0.025 ^b^	0.07				
giberrellin A3	0.014 ^b^	0.01				
giberrellin A4	0.035 ^b^	0.01				
giberrellin A7	0.014 ^b^	0				

^a^ lower limit of detection <5 ng ml^-1^.

^b^ average of duplicate determinations.

Trace albeit extremely low concentrations of indoleacetic, absicic and jasmonic acid, methyl jasmonate, and gibberellin A1, A3, A4, and A7 were detected in catering waste leachate. The corresponding levels in frass were barely detectable and approximately 10 to 100-fold less than that found in catering waste leachate (**Table 1)**.

The N:P_2_0_5_:K_2_O percent dry matter average of BSFL processed catering waste residues was 3.27 ± 0.21 (±1SD, n = 3), 3.14 ± 0.08 (±1SD, n = 3) and 4.45 ±-0.38 (±1 SD, n = 3), respectively. Its calculated average N:P_2_0_5_:K_2_O oxide ratio rounded to the nearest tenth was 1.0:1.0:1.3.

The N:P_2_0_5_:K_2_O percent dry matter average of frass was markedly lower averaging 0.09 ± 0.07 (±1 SD, n = 5), 0.04 ± 0.03 (±1 SD, n = 5) and 0.32 ± 0.23 (±1 SD, n = 5), respectively. Its calculated average N:P_2_0_5_:K_2_O oxide ratio (rounded to the nearest tenth) was 1.0:0.4:3.5.

**Table 2** contrasts the growth of winter wheat berry seedlings grown in potting soil amended with frass to that of seedlings grown in unamended (control) potting soil. Frass accelerated the growth of seedlings as evidenced by an average 11% increase in total upper plant mass relative to that of seedlings grown in unamended potting soil concomitant with a statistically significant 11% increase in the average shoot length of the frass-treated plants.

**Table 2:** Average 30 day upper plant mass and shoot length growth rate of winter wheat berry seedlings grown in potting soil treated with BSFL frass *versus* unamended (control) potting soil.

	Average Upper Plant Mass (g/pot)	Average Shoot Length (cm)
BSFL frass–treated soil test set	13.9 ± 1.4 (±1 SD, n = 4)	31.1 ± 3.7 (±1 SD, n = 137) ^a^
Control–untreated soil test set	12.5 ± 1.7 (±1 SD, n = 4)	28.1 ± 4.2 (±1SD, n = 137) ^a^
Percent growth relative to control test set	+11%	+11%
One tailed t-test	(0.17)	(1.5 x 10^−9^) ^a^

^a^ Statistically significant findings.

**Fig 1:**
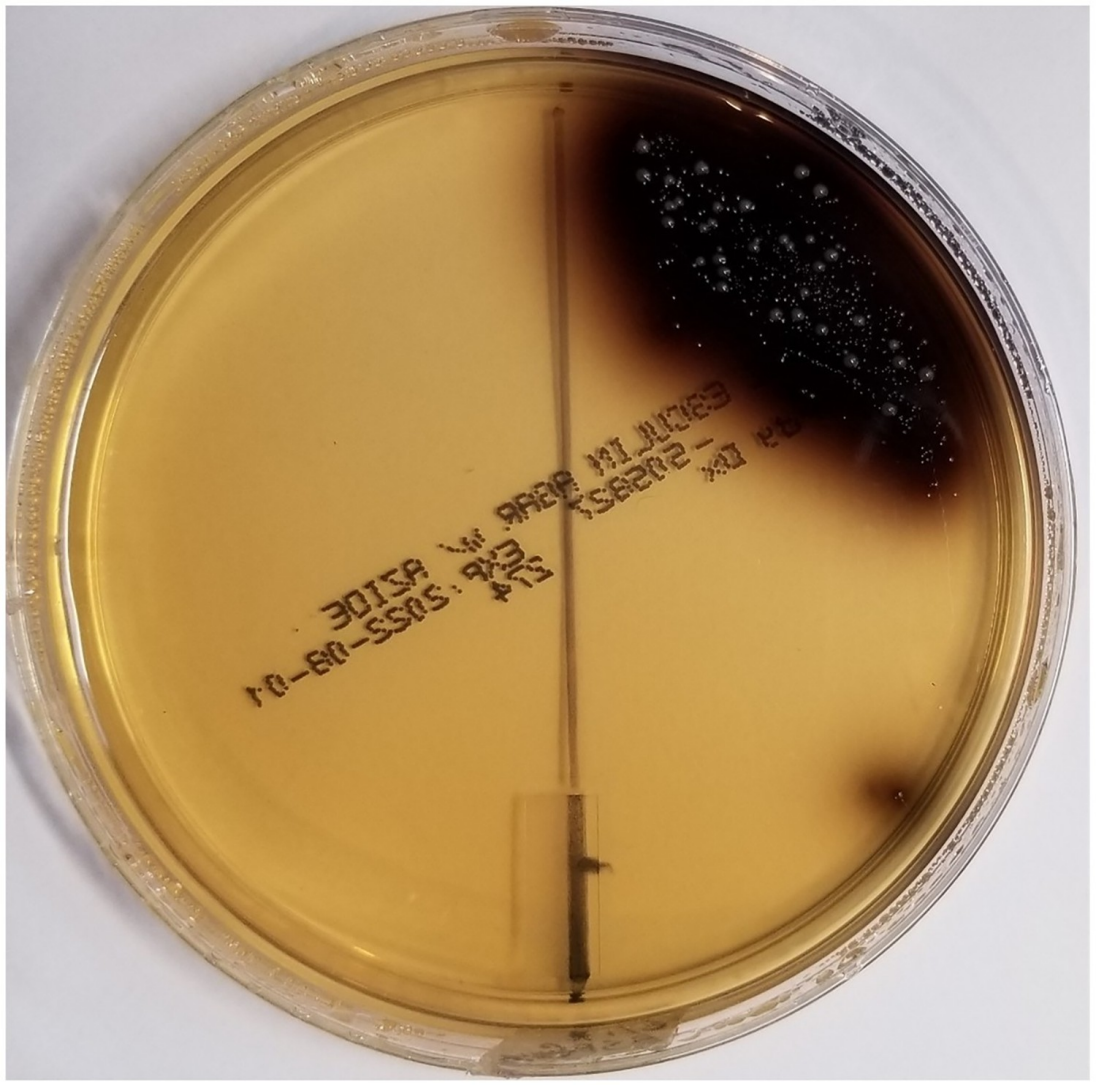
BEA agar plate assay confirming the presence of viable *Enterococci* in frass recovered from BSFL. Right side of plate–frass test results; Left side of plate–test results run on water (control) used in suspending the larvae before collection of BSFL frass. Note detection of multiple colonies and positive iron esculetin coloration on right side of plate and the absence of colonies on the left (control) side of the plate.

Numerous microbial colonies characteristic of the genus *Enterococcus* grew out on BEA agar petri dish plates streaked with larval frass recovered directly from larvae washed free of contaminating waste feedstock (**Fig 1**).

**Fig 2:**
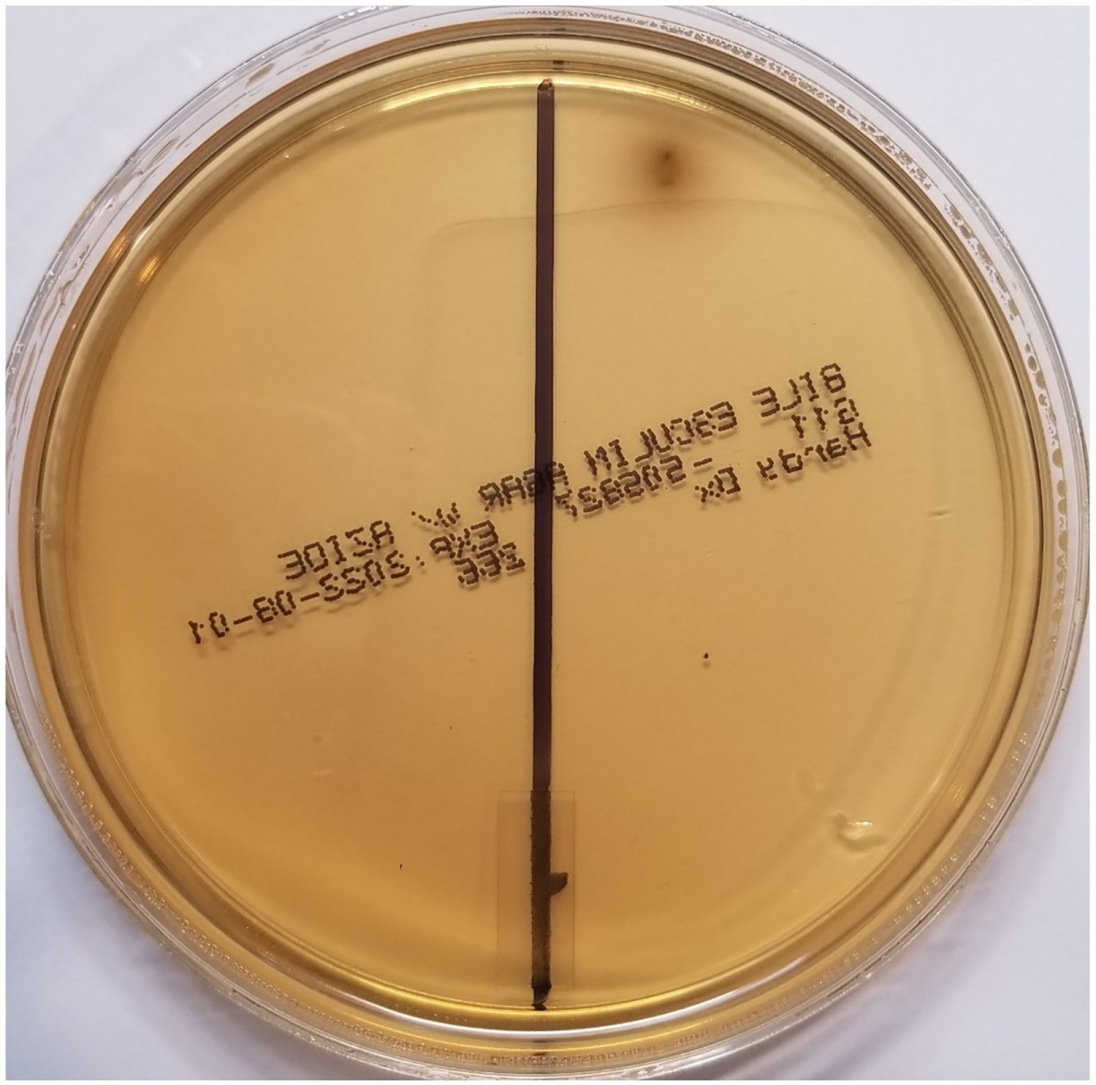
BEA agar plate assay test results for viable *Enterococci* sampled immediately after suspension of washed BSFL in sterile water. Right side of plate–test results immediately after suspending the larvae in water (T = 0); Left side of plate—test results run on water (control) used in suspending the larvae before collection of BSFL frass.

**Fig 2** shows the results of plating out a sample from washed BSFL suspended in sterile water drawn immediately after the larvae were suspended in solution. Only two positive colonies can be seen on the plate as opposed to the numerous colonies seen in **Fig 1**. These results indicate that the preponderance of *Enterococci* detected as in **Fig 1** were from their frass.

## Discussion

To understand how BSFL frass confers to soil plant growth promoting activity, it is important to examine not only its nutrient N:P_2_0_5_:K_2_O percent dry matter average, biogenic amine and phytohormone content, but also to test whether rhizobacteria are present in the frass. Gärttling and Schulz [1] have pointed out that “In the context of black soldier fly (BSF) rearing, often the residues from production–mainly faeces but also undigested substrate–are addressed as frass in a broader sense.” Klammsteiner et al. [20] also noted the same issue. Lopes, Yong and Lalander [2] in yet another review commented on the instability of BSFL processed waste residues, both positive and negative effects on plant growth and differences in C/N ratios and N:P_2_0_5_:K_2_O percent dry matter averages. In all of these studies investigators have principally focused on the N:P_2_0_5_:K_2_O percent dry matter average of frass, inferring that frass is serving as a fertilizer in conferring to treated soils plant growth promoting activity. Very little attention appears to have been given to the possible contribution of rhizobacteria in conferring to frass its plant growth promoting properties.

In this study frass was separated free of contaminating waste feedstock, insect exuviae and particulate matter. It was then examined with regard to: (i) its N:P_2_0_5_:K_2_O percent dry matter average which if significant could be serving as a source of nutrient fertilizer; (ii) its phytohormone content, particularly its indole acetic acid and/or gibberellin content, which if present in sufficient concentrations could cause a growth spurt due to the action of phytohormones inducing stem cell elongation and/or an overall increases in the plant’s aerial mass [21–23]; (iii) its biogenic amine content which if present in significant concentrations could confer an advantage in plant growth to treated plants by alleviating abiotic stresses [24–27]; (iv) whether or not upon amendment into soil it confers to soil plant growth promoting activity; and (v) whether or not *Enterococci* are present in the frass which if present could account for its plant growth promoting activity on amendment into soil [12–14].

The N:P_2_0_5_:K_2_O percent dry matter average and calculated oxide ratio of BSFL processed catering waste residues in this study at 3.3:3.1:4.5 and at 1.0:1.0:1.3 (rounded to the nearest tenth), respectively, is in reasonably good agreement with the data sets collated and the calculated average oxide ratio of 1.0:0.9:1.1 summarized by Gärttling and Schulz [1]. The much lower N:P_2_0_5_:K_2_O percent dry matter average of frass at 0.09:0.04:0.32 (reported to the nearest hundredth), respectively, only trace concentrations of phytohormones, and complete absence of biogenic amines in the frass makes it highly unlikely that any of these constituents account for the plant growth promoting activity detected in frass following its amendment in soil.

Since numerous colonies of *Enterococci* were detected in frass collected directly from larvae suspended in sterile water, these latter results confirm the hypothesis that viable *Enterococci* from the larval gut are excreted into frass. In drawing this conclusion, it should be noted that microorganisms in the phylum Firmicutes and the genus *Clostridium* are present and have been identified in food waste processed by BSFL [28]. Because of these latter findings, to definitively rule out cross residual waste contamination and carryover of *Enterococci*, larvae were first washed free of contaminating waste feedstock in copious quantities of sterile water before screening the larval suspensions for *Enterococci*. The paucity of *Enterococci* colonies growing out on samples drawn immediately after suspending the washed larvae in sterile water (T = 0) rules out cross contamination (cf., **Fig 2**).

There is a considerable amount of evidence that microbes confer plant growth promoting properties to leftover wastes amended back into soil. Several microorganisms from the phylum Firmicutes, including the genus *Enterococcus*, and the genus *Clostridium* [28], have previously been detected in BSFL processed waste and shown to promote the growth of barley and mung bean plants, to enhance seed germination rates for beet, barley, wheat, red radish, cucumber and tomato plants [29, 30], and Pakchoi (*Brassica rapa* L.) [31]. Sasmita et al. [32] and Pakpahan et al. [7] have also demonstrated a link between the expression of plant growth promoting activity and liquid BSFL extracts recovered from BSFL processed vegetal, fruit and dairy waste feedstocks. Microorganisms identified and capable of fixing N in soil, P solubilizers and phytohormone producers were associated with *Pseudomonas spp*., *Bacillus spp*., *Trichoderma spp*., *Streptomyces spp*., *Azotobacter spp*., and *Azospirillum spp*.

The demonstration that viable *Enterococci* pass from the larval gut into larval frass as shown in this study is a necessary first step in justifying further in-depth research aimed at identifying which specific strains of *Enterococci*, and other rhizobacteria, are present in frass that have the capability of conferring to frass plant growth promoting activity upon colonizing the rhizosphere of receptive plants [9, 15, 16]. In this regard, among the microbiota recently identified in significant abundance in the larval gut, two species, *Enterococcus casseliflavus* and *Enterococcus faecium* (11), are of particular interest as possible candidates involved in conferring to frass its plant growth promoting activity. Both have previously been shown to promote the growth of plants upon amendment into soil [12, 13]. Given the growing awareness and application of rhizobacteria as an effective means of boosting agricultural crop yields, further research aimed at identifying more broadly the relative abundance, diversity and characteristics of rhizobacteria, including which *Enterococci spp*. are present in frass exhibiting rhizobacterial activity, is warranted in light of the findings in this study.

## Supporting information

S1 File (PDF)

S2 File (PDF)

S3 File (XLS)
