# Supplementary material for: A biochemical analysis of Black Soldier fly (Hermetia illucens) larval frass plant growth promoting activity
Source: PLoS One. 2023 Jul 19;18(7):e0288913. doi: 10.1371/journal.pone.0288913 (PMC10355398; doi:10.1371/journal.pone.0288913)
Supplement: S2 File — (PDF) [file pone.0288913.s002.pdf]

www.lifeasible.com
Lifeasible
17 Ramsey Rd. Suite 209
Shirley, NY 11967

PROJECT REPORT

Plant Hormone Analysis for Biomass Fermented Waste

Project Number LZBD12292005

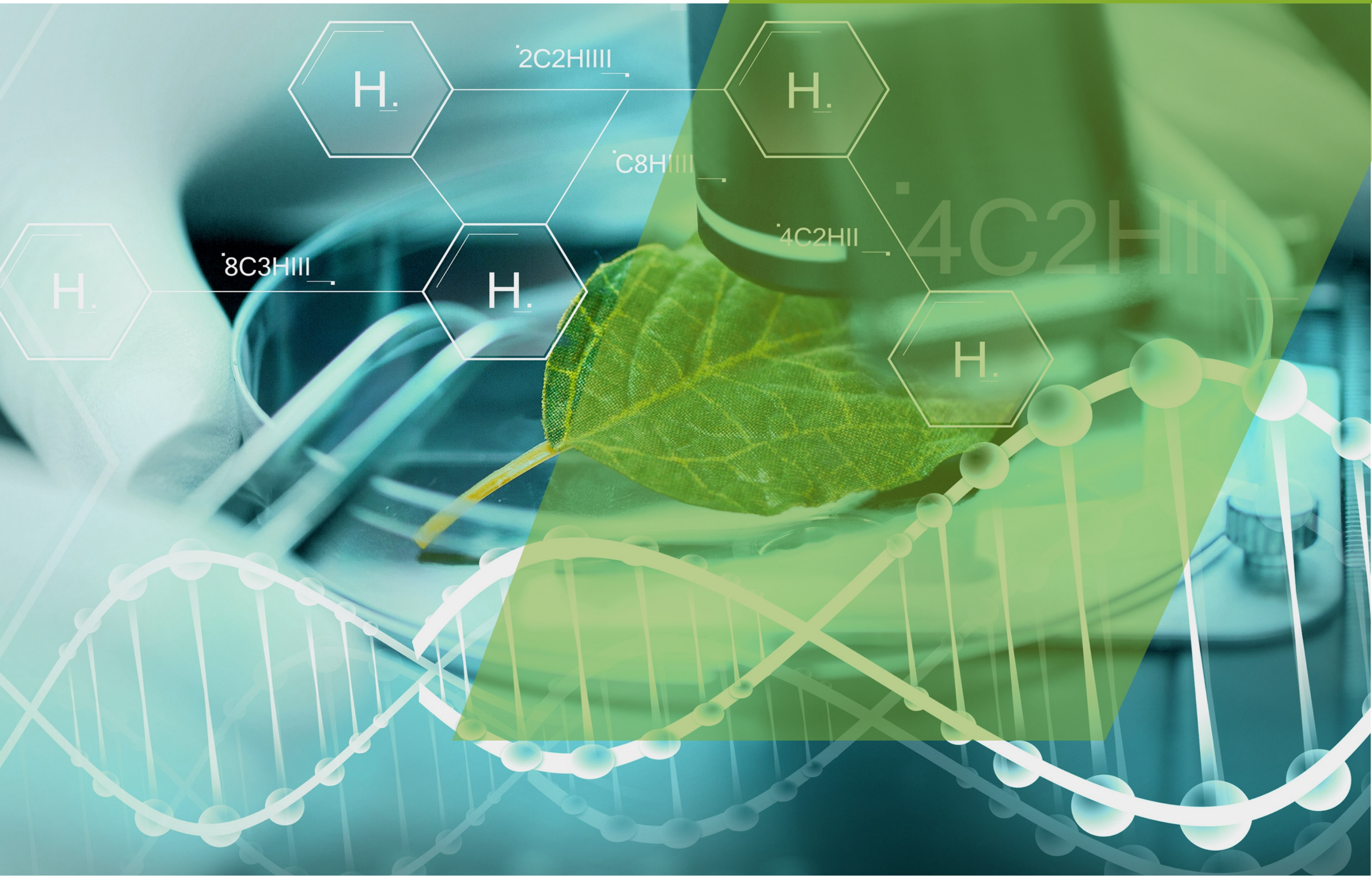

Lifeasible

Report generated by
Rujane Zhang Ph.D., R&D Manager

Report Reviewed by
Miao Li Ph.D., Project Manager

On February 8 2021

Table of Content

1 Project Information.....1

2 Methods and Materials..... 2

2.1 Samples.....2

2.2 Reagents and Consumables.....2

2.3 Equipment.....3

2.4 Methods.....3

2.4.1 Preparation of Standard Solution of Plant Hormones.....3

2.4.2 Phytohormones Extraction.....3

2.5 HPLC-MS/MS Program.....4

2.5.1 HPLC Conditions for Phytohormones.....4

2.5.2 Mass Spectrometry Parameter for Phytohormones.....4

3 Results.....6

3.1 Calibration Curves.....6

3.2 Results.....6

4 Conclusions and Discussions.....7

1 Project Information

The service quotation [Number LZBD12292005] is for quantification analysis of the plant hormones for biomass fermented waste using electrospray ionization-high-performance liquid chromatography tandem mass spectrometry (ESI-HPLC-MS/MS). The plant hormones include indole-3-acetic acid (IAA), jasmonic acid (JA), methyl jasmonate (Me-JA), abscisic acid (ABA), gibberellic acid 1 (GA1), gibberellic acid 3 (GA3), gibberellic acid 4 (GA4), and gibberellic acid 7 (GA7). Two samples derived from biomass fermented waste are provided by customer. We have successfully finished the project.

2 Methods and Materials

2.1 Samples

- Two samples of BSF101E and FS101C are provided by customer.

Sample Label	Sample NO.	Form
BSF101E	B1	Liquid
FS101C	B2	Liquid

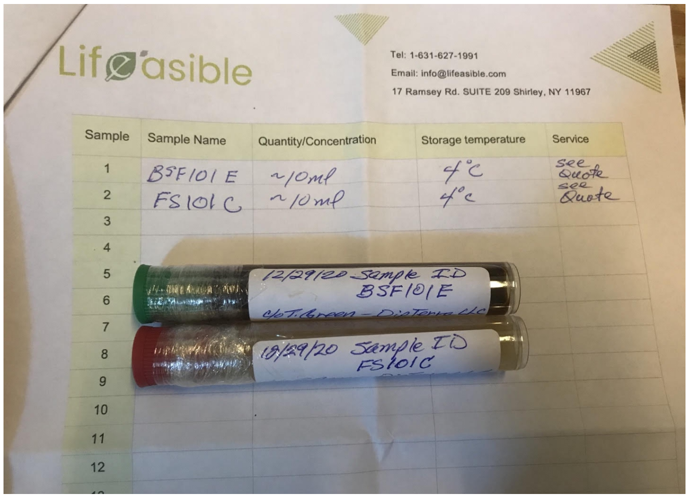

2.2 Reagents and Consumables

- Indole acetic acid (IAA) standard (Sigma-aldrich)
- Abscisic acid (ABA) standard (Sigma-aldrich)
- Jasmonic acid (JA) standard (Sigma-aldrich)
- Methyl jasmonate (Me-JA) standard (Sigma-aldrich)
- Gibberellin acid 1 (GA1) standard (TRC)
- Gibberellin acid 3 (GA3) standard (Sigma-aldrich)
- Gibberellic acid 4 (GA4) standard (Sigma-aldrich)
- Gibberellic acid 7 (GA7) standard (TRC)
- Deuterium-ABA standard (Sigma-aldrich)
- Deuterium -IAA standard (Sigma-aldrich)
- Chromatographic methanol (Merck)

- Analytical acetonitrile (Merck)
- Chromatographic formic acid (Merck)
- C18 Queschers packing (Anpel)
- PSA Queschers packing (Anpel)

2.3 Equipment

- AGLIENT1290 HPLC system (AGLIENT)
- AB SCIEX-6500 Qtrap MS/MS (Applied Biosystems)

2.4 Methods

2.4.1 Preparation of Standard Solution of Plant Hormones

1) 984 μL methanol was added into a 1.5 mL centrifuge tube and 500 μg/mL standard reserve solution of 2 μL for each hormone was added. The solution was well mixed prepared as the mother solution with final concentration of 1 μg/mL.
2) 996 μL methanol was added into a 1.5 mL centrifuge tube and 2 μL of 500 μg/mL internal standard reserve solution of each hormone was added. The solution was well mixed and prepared as the mother solution with final concentration of 1 μg/ mL.
3) Methanol of 989.9 μL, 989.8 μL, 989.5 μL, 988 μL, 985 μL, 970 μL, 940 μL, 790 μL was added into a 1.5 mL centrifuge tube, and the mother solution prepared in step (1) was added into the methanol solution in sequence of 0.1 μL, 0.2 μL, 0.5 μL, 2 μL, 5 μL, 20 μL, 50 μL, 200 μL, respectively. Then add 10 mL the internal standard in step (2) per tube to make standard curvature solution with final concentration 0.1 ng/ mL, 0.2 ng/mL, 0.5 ng/mL, 2 ng/mL, 5 ng/mL, 20 ng/mL, 50 ng/mL, 200 ng/mL and containing 10 ng/mL internal standard.

2.4.2 Phytohormones Extraction

1) Shake the liquid sample thoroughly, accurately take 5 mL into the tube, and add 10 mL dichloromethane and 2 μL internal standard mother solution.
2) The sample solution was briefly vortexed and then kept at 4 °C overnight.
3) Following centrifugation of 5 min at 12,000 g at 4 °C, the lower dichloromethane phase was taken.
4) Add 35 mg C18 packing, shake violently for 30 s, centrifuge at 10,000 g for 5 min, and get the supernatant.
5) The supernatant was dried by nitrogen and then dissolved in 200 μL methanol.

6) After filtration through a membrane filter (0.22 μm), 2 μL extract was injected onto a C18 column mounted on an analytical HPLC system (AGLIENT) equipped with AB SCIEX-6500 Qtrap MS/MS.

2.5 HPLC-MS/MS Program

2.5.1 HPLC Conditions for Phytohormones

Column: Poroshell 120 SB-C18 reversed phase column (2.1 mm x 150 mm, 2.7 μm);

Column temperature: 30 °C;

Mobile phase: A:B = (Methanol / 0.1% formic acid): (water / 0.1% formic acid);

Gradient Parameters of HPLC:

Table 1 Gradient Parameters of HPLC for Phytohormones

Time (min)	Flow Rate (mL/min)	A%
0.1	0.3	20
1-3	0.3	Increment to 50
3-9	0.3	Increment to 80
9-10.5	0.3	80
10.5-10.6	0.3	Decrease to 20
10.6-13.5	0.3	20

Injection volume: 2 μL.

2.5.2 Mass Spectrometry Parameter for Phytohormones

Ionization mode: ESI positive and negative ion mode monitoring;

Scan type: MRM;

Air curtain gas: 15 psi;

Spray voltage: +4500 v, -4000 V;

Atomizing gas pressure: 65 psi;

Auxiliary gas pressure: 70 psi;

Atomization temperature: 400 °C.

Table 2 Selected Reaction Monitoring Conditions for Protonated or Deprotonated Phytohormones ($[M+H]^+$or$[M-H]^-$)

Name	Polarity	Parent Ion (m/z)	Daughter Ion (m/z)	Declustering Voltage (V)	Collision Energy (V)
IAA	+	176.2	129.8*/102.9	65	12/42
JA	-	209.2	58.9*	-54	-16
MeJA	+	225.1	151.1/193.1/133*	50	16/10/18
ABA	-	263.1	153.0*/204.2	-60	-14/-27
GA1	-	347.2	259.2*/273.0	-25	-28/-16
GA3	-	345.2	143.0/239.0*	-80	-30/-33
GA4	-	331.1	243.2*/213.1	-131	-24/-39
GA7	-	329.2	223.2/241.1*	-89	-38/-22

*Quantitative ions

3 Results

3.1 Calibration Curves

Each calibration curve was drawn by AB SCIEX-6500 Qtrap MS/MS instrument according to data of STD1-8. (Raw data see other files).

3.2 Results

The detection concentrations of phytohormones hormones in sample were calculated with the calibration cures. In summary, the content (ng/mL) of phytohormones was expressed as quantity of per volume (Raw and calculated data see other files). The contents of IAA in two samples (0.36 ng/mL vs 50.39 ng/mL) were the highest.

4 Conclusions and Discussions

Here we successfully performed ESI-HPLC-MS/MS analysis for plant hormones of 2 samples – BSF101E and FS101C - provided by customer. The concentration (ng/mL) of each item tested in each sample is listed in a summary table below (Table 3).

Table 3. Summary of Contents

Component Name	BSF101E (ng/mL)	FS101C (ng/mL)
IAA	0.36	50.39
ABA	0.039	5.37
JA	0.18	0.99
Me-JA	0.020	0.031
GA1	0.068	0.025
GA3	0.010	0.014
GA4	0.0087	0.035
GA7	0.00080	0.014

– End of Report –

Lifeasible

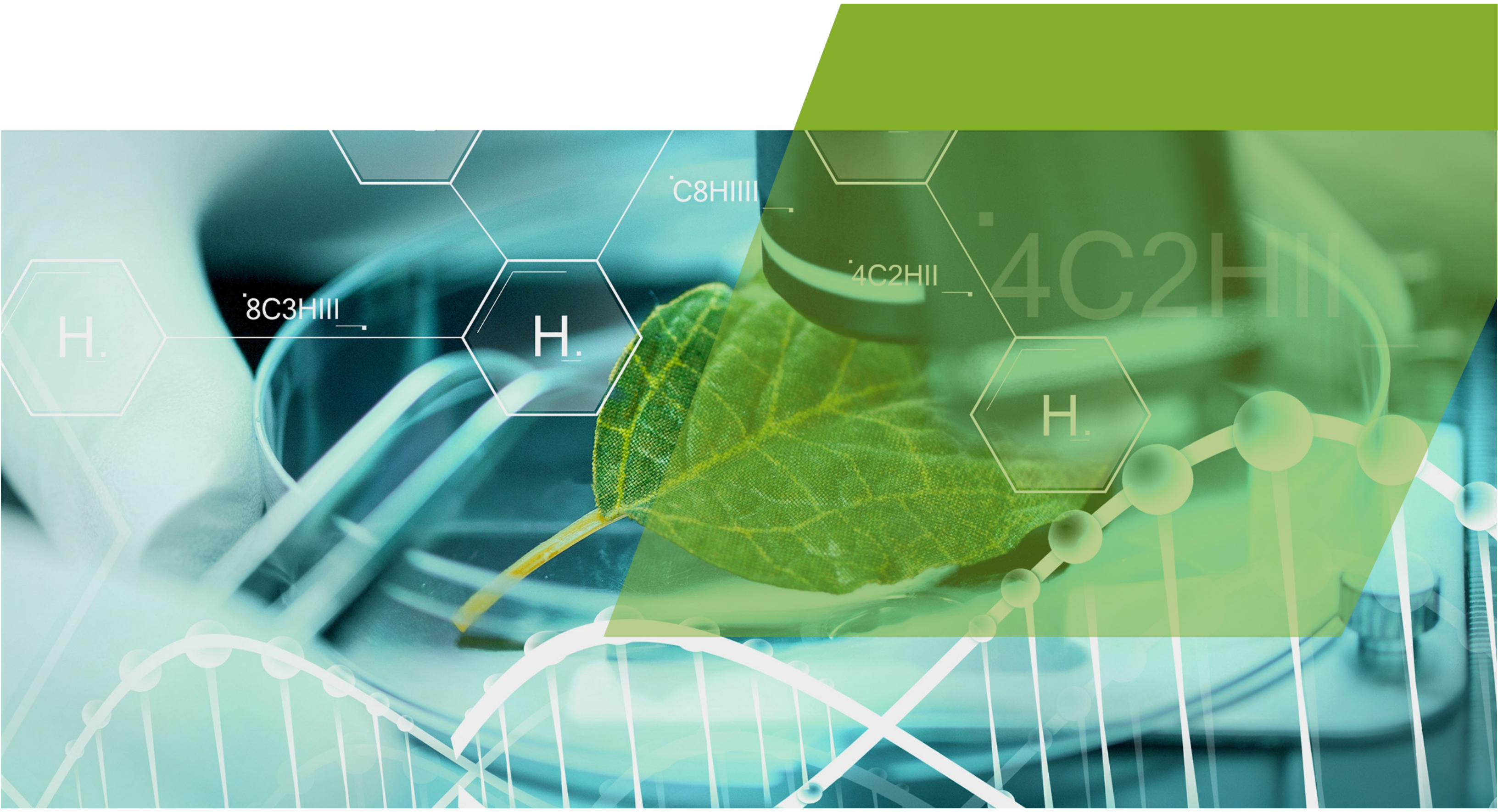

CONTACT US

17 Ramsey Rd. Suite 209, SHirley, NY 11967

TEL: 1-631-627-1991

FAX: 1-631-910-2166

EMAIL: info@lifeasible.com

Web: https://www.lifeasible.com
